# Investigating the Regulation of Ribosomal Protein S6 Kinase 1 by CoAlation

**DOI:** 10.3390/ijms25168747

**Published:** 2024-08-11

**Authors:** Oksana Malanchuk, Anna Bdzhola, Sergii Palchevskyi, Volodymyr Bdzhola, Peng Chai, Olivier E. Pardo, Michael J. Seckl, Adrija Banerjee, Sew Yeu Peak-Chew, Mark Skehel, Lalitha Guruprasad, Alexander Zhyvoloup, Ivan Gout, Valeriy Filonenko

**Affiliations:** 1 Department of Structural and Molecular Biology, University College London, London WC1E 6BT, UK; o.malanchuk@ucl.ac.uk (O.M.); a.zhyvoloup@ucl.ac.uk (A.Z.); 2 Department of Cell Signalling, Institute of Molecular Biology and Genetics, National Academy of Sciences of Ukraine, 03143 Kyiv, Ukraine; a.v.bdzhola@imbg.org.ua (A.B.); s.s.palchevskyy@imbg.org.ua (S.P.); v.g.bdzhola@imbg.org.ua (V.B.); 3 Division of Cancer, Imperial College London, Du Cane Road, London W12 0NN, UK; p.chai20@imperial.ac.uk (P.C.); o.pardo@imperial.ac.uk (O.E.P.); m.seckl@imperial.ac.uk (M.J.S.); 4 School of Chemistry, University of Hyderabad, Hyderabad 500 046, India; adrija.swity97@gmail.com (A.B.); lalitha.guruprasad@gmail.com (L.G.); 5 Biological Mass Spectrometry & Proteomics Cell Biology, MRC Laboratory of Molecular Biology, Francis Crick Avenue, Trumpington, Cambridge CB2 0QH, UK; spc@mrc-lmb.cam.ac.uk; 6 The Francis Crick Institute, 1 Midland Road, London NW1 1AT, UK; mark.skehel@crick.ac.uk

**Keywords:** kinase regulation, oxidative stress, p70S6K1, coenzyme A, protein CoAlation, post-translational modifications, cell signalling

## Abstract

Ribosomal protein S6 kinases belong to a family of highly conserved enzymes in eukaryotes that regulate cell growth, proliferation, survival, and the stress response. It is well established that the activation and downstream signalling of p70S6Ks involve multiple phosphorylation events by key regulators of cell growth, survival, and energy metabolism. Here, we report for the first time the covalent modification of p70S6K1 by coenzyme A (CoA) in response to oxidative stress, which regulates its kinase activity. The site of CoA binding (CoAlation) was mapped by mass spectrometry to cysteine 217 (Cys217), located in the kinase activation loop and only one amino acid away from the tripeptide DFG motif, which facilitates ATP-binding. The CoAlation of recombinant p70S6K1 was demonstrated in vitro and was shown to inhibit its kinase activity. Our molecular docking and dynamics analysis revealed the most likely mode for CoA binding to p70S6K1. This mechanism involves the non-covalent binding of the CoA ADP moiety to the p70S6K1 nucleotide-binding pocket, positioning the CoA thiol group in close proximity to form a covalent bond with the surface-exposed Cys217 residue. These findings support a “dual anchor” mechanism for protein kinase inhibition by CoAlation in cellular response to oxidative stress. Furthermore, the inhibition of S6K1 by CoAlation may open new avenues for developing novel inhibitors.

## 1. Introduction

Ribosomal S6 kinases are divided into two distinct families of Ser/Thr kinases in mammalian cells, termed p90RSK and p70S6K. Each kinase exhibits different mechanisms of activation and downstream signalling, as well as cellular responses. p90RSKs are activated via the Ras-mitogen-activated protein kinase (MAPK) pathway and regulate cell proliferation, differentiation, and survival via the phosphorylation of immediate early gene products, translational regulators, including the ribosomal S6 protein [1,2]. In contrast, p70S6Ks function downstream of the mammalian target of rapamycin (mTOR), phosphoinositide 3-kinase (PI3K) and/or the Ras-MAPK signalling pathway. The substrates of p70S6Ks include regulators of mRNA processing, protein synthesis, glucose homeostasis, cell growth, and survival [3].

There are two members of S6Ks known as S6K1 and S6K2. They are encoded by *RPS6KB1* on chromosome 17 and *RPS6KB2* on chromosome 11, respectively. Several kinase isoforms are produced from both genes through the use of alternative translation start sites and alternative splicing. The most commonly expressed and studied isoforms are: p70S6K1 and p85S6K1 for S6K1, along with p54S6K (S6KβII) and p56S6K (S6KβI) for S6K2 [4,5,6,7]. Furthermore, we recently characterized a novel p60 isoform of S6K1 with a regulatory mechanism distinct from that of p70S6K1 and p85S6K1 [8].

The larger isoforms of S6K1 and S6K2 possess functional nuclear localisation signals in their N-terminal extensions, which target them to the nucleus [9]. In addition, S6K1 and S6K2 share a modular organisation homologous to that of many Ser/Thr kinases, consisting of an N-terminal regulatory region (NTD), a kinase domain, followed by a kinase extension domain and C-terminal regulatory region (CTD). They are members of the AGC family of Ser/Thr kinases, which also includes PKA, Akt (PKB), PKCs, PKG, PDK-1, SGK, and p90RSK [10].

p70S6K1 contains eight Ser/Thr phosphorylation sites, which regulate a series of stepwise conformational changes that result in kinase activation. It is now well established that phosphorylation of Thr229 (the T-loop site), Thr389 (the hydrophobic motif site), and Ser371 (the turn motif site) are required for full p70S6K1 activation. In addition to phosphorylation, p70S6K1 is regulated by other post-translational modifications (PTMs) in cellular response to mitogenic stimuli, nutrient availability, and stress responses. The availability and use of mouse, fly and worm models revealed that p70S6K1 is an important regulator of cell size, growth, and cellular metabolism [11,12,13,14]. These diverse functions cannot be attributed solely to the phosphorylation of ribosomal protein S6 (rpS6). Multiple additional downstream substrates and regulators have been discovered, including key players in mRNA processing and translation, ribosome biogenesis, cellular metabolism, and survival. The dysregulation of S6K1-dependent signalling has been observed in various human pathologies, including cancer, obesity, and diabetes, making it an attractive target for drug discovery [15,16,17].

Coenzyme A (CoA) is a ubiquitous metabolic cofactor essential for all living cells [18]. CoA biosynthesis is a highly conserved pathway involving the sequential conjugation of pantothenate (vitamin B5), cysteine, and adenosine triphosphate (ATP) [19]. The presence of a highly reactive thiol group at the tip of its pantetheine tail provides CoA with the capacity to function as a cofactor in numerous biochemical reactions and to form thioester compounds found within a plethora of metabolic pathways [18]. In this way, CoA occupies a relatively unique position amongst metabolic cofactors at a strategic crossroads between many major branches of metabolism including fatty acid oxidation, amino acid metabolism and the biosynthesis of ketone bodies, cholesterol, ATP, and acetylcholine. Moreover, CoA also plays roles in the regulation of chromatin maintenance, gene transcription, and cellular metabolism via its acyl-derivatives which are used as substrates for the process of protein acetylation, a PTM used to regulate gene expression as well as signalling and metabolic pathways [20]. Whilst the roles of CoA in forming metabolically active thioester derivatives are well defined, its roles as a low molecular weight (LMW) thiol in cellular antioxidant defence mechanisms are comparatively less studied [21]. An association between the mTOR/S6K signalling pathway and energy metabolism through CoA and its thioester derivatives was also reported [22].

Cells contain multiple antioxidant defence systems which protect major cellular macromolecules from irreversible damage caused by endogenously produced or exogenous reactive oxygen species (ROS) [23]. LMW thiols are important players of the antioxidant defence, and glutathione is one of the most studied and potent antioxidants [24]. Recently, CoA was found to function as a major antioxidant in prokaryotic and eukaryotic cells exposed to oxidative or metabolic stress [25,26]. This discovery was made possible by the development of novel reagents and methodologies, including (a) highly specific anti-CoA monoclonal antibodies, which work efficiently in various immunological assays; (b) mass spectrometry-based methodology for the identification of CoAlated proteins, and (c) in vitro CoAlation and deCoAlation assays [27]. These advances have led to the identification of a novel PTM, now termed protein CoAlation. CoAlation involves the formation of a mixed disulphide bond between CoA and a redox-sensitive cysteine residue modified under oxidative stress. 

Subsequent studies revealed that protein CoAlation is a reversible and widespread PTM, which occurs in mammalian cells and tissues, as well as other model organisms such as bacteria, yeast, worms, and amoeba upon exposure to oxidative or metabolic stress [25,26]. To date, over 2100 CoAlated proteins have been identified in eukaryotic and prokaryotic cells. The bioinformatic analyses of the protein CoAlome indicate that CoA-modified proteins are predominantly involved in metabolic processes, as well as stress responses and protein synthesis [28]. The identification of CoAlated proteins and the development of in vitro CoAlation assays have prompted investigations into how the covalent modification of proteins by CoA influences their cellular function [21]. Biochemical, biophysical, crystallographic, cell, and molecular biology studies have revealed that CoAlation can modulate the activity and subcellular localization of CoA-modified proteins [28]. Moreover, CoAlation was shown to protect oxidized cysteine residues from irreversible overoxidation and induce extensive conformational changes [29].

More than 35 protein kinases have been found to be CoAlated in mammalian cells and tissues under oxidative stress. Our attention was focused on p70S6K1, which was found to be CoAlated on Cys217 in HEK293/Pank1β cells treated with diamide. Cys217 is located in the kinase activation loop and only one amino acid away from the tripeptide DFG motif. We have recently reported a novel and unique mode of Aurora A kinase inhibition by CoA. This mechanism involves the selective binding of the CoA ADP moiety to the p70S6K1 nucleotide-binding pocket, followed by covalent bond formation between the pantetheine thiol and Cys290 located in the activation loop [29]. By analogy to the covalent inhibition of Aurora A kinase by CoA, we employed a combination of biochemical, biophysical, cell biology, and bioinformatic approaches to study the effect of Cys217-inuced p70S6K1 regulation by CoAlation at Cys217 under oxidative stress.

## 2. Results

### 2.1. p70S6K1 Is CoAlated at Cys217 in HEK293/Pank1β Cells Treated with Diamide

In this study, we used HEK293/Pank1β cells with the stable overexpression of pantothenate kinase 1β (Pank1β), which is the major rate-limiting enzyme in the CoA biosynthetic pathway. The stable overexpression of Pank1β was shown to significantly induce CoA biosynthesis in COS-7 cells [30]. It was demonstrated that HEK293/Pank1β cells produce levels of CoA comparable to primary cardiomyocytes, rat heart, liver, and kidney cells [25]. We have also shown that the extent of protein CoAlation is determined by the level of CoA in cells and tissues [25].

**Figure 1:**
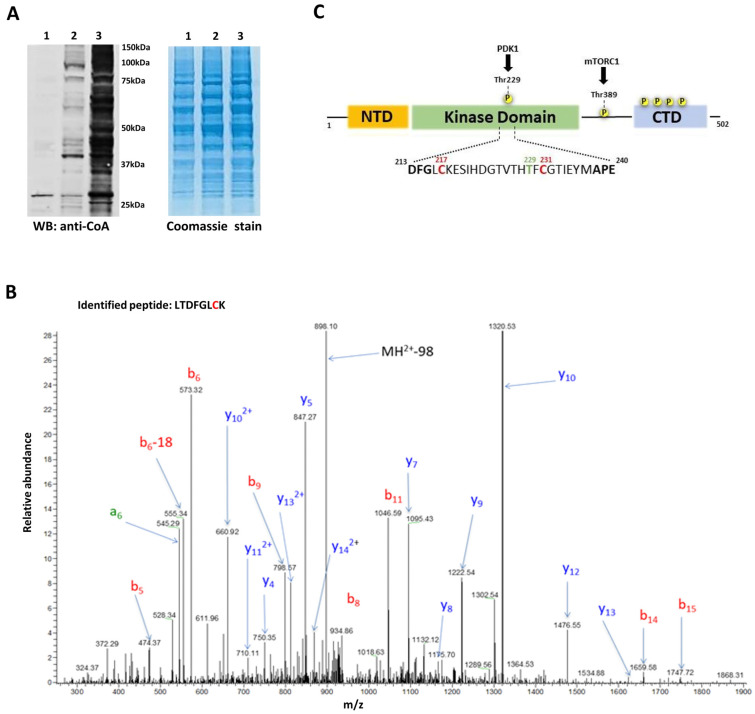
LC-MS/MS identification of p70S6K1 CoAlation in HEK293/Pank1β cells treated with diamide. (**A**) Anti-CoA Western blot reveals extensive covalent modification of cellular proteins by CoA in HEK293/Pank1β cells treated with 500 µM H_2_O_2_ (lane 2) and 500 µM diamide (lane 3) for 30 min, compared to untreated control cells (lane 1). The experiment was repeated at least three times with similar results obtained. (**B**) Liquid chromatography–tandem mass spectroscopy (LC-MS/MS) spectrum of a CoAlated peptide (LTDFGLC*K), corresponding to p70S6K1. (**C**) Schematic domain structure of p70S6K1. CoAlated Cys217 is located in the activation loop, as well as Cys231. The activation loop (annotated) starts with the DFG motif and ends with the APE motif. Thr229 is phosphorylated by PDK1, which is critical for S6K1 activation.

Here, we found that protein CoAlation is strongly induced in HEK293/Pank1β cells treated with 500 µM H_2_O_2_ or 500 µM diamide (Figure 1A). To identify CoAlated proteins in diamide-treated cells, which showed extensive CoAlation, we used in-house developed monoclonal antibodies against CoA [27] and MS-based methodology [25]. Many kinases were found to be CoAlated, with substrate specificities ranging from proteins to lipids, carbohydrates, and nucleotides. Our interest in studying the regulation of p70S6K1 by CoA was based on the following criteria. First, the CoAlated peptide corresponding to p70S6K1 was found in HEK293/Pank1β cells treated with diamide. Figure 1B shows the LC–MS/MS spectra of the cysteine-containing CoAlated peptide (LTDFGLC*K) derived from p70S6K1. Second, CoAlated Cys217 is located in the activation loop and one amino acid adrift from the DFG motif, which is required for kinase catalytic activity. Furthermore, the site of PDK1 phosphorylation (Thr229), which is critical for p70S6K1 activation, is also located in the activation loop, in close proximity to Cys217. Therefore, it is possible that CoAlation at Cys217 might regulate p70S6K1 activity by modulating the state of PDK1 phosphorylation/dephosphorylation at Thr229. The position of Cys217 relative to the overall structure of p70S6K1 is schematically presented in Figure 1C. Finally, by analogy to Aurora A kinase/CoA interaction, we anticipated the inhibitory effect of CoAlation on the activity, downstream signalling, and function of p70S6K1 under oxidative stress [29].

### 2.2. Transiently Overexpressed EE-p70S6K1 Is CoAlated in HEK293/Pank1β Cells Exposed to Oxidative Stress

**Figure 2:**
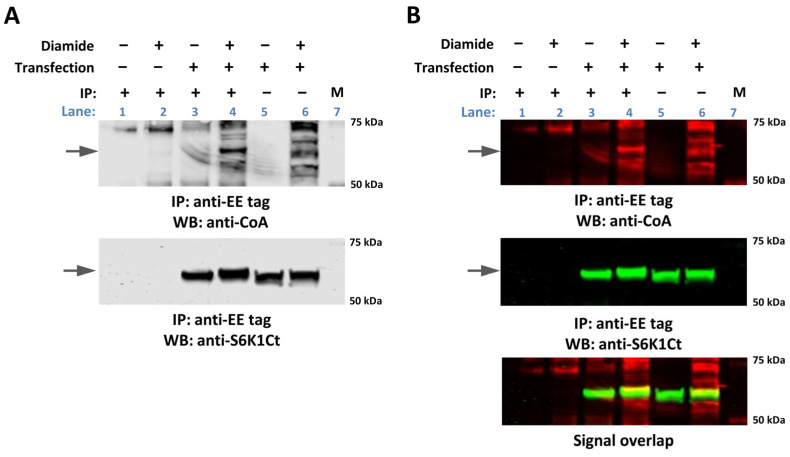
Transiently overexpressed EE-p70S6K1 is CoAlated in HEK293/Pank1β cells exposed to oxidative stress. (**A**) Transiently overexpressed EE-p70S6K1 was pulled down from the lysates of untreated and diamide-treated cells with anti-EE mAb. Total cell lysates and immunoprecipitated proteins were examined by Western blotting (WB) with anti-CoA and anti-S6K1 antibodies. The CoAlation of immunoprecipitated EE-S6K1 in diamide-treated cells was detected by WB analysis with anti-CoA mAb (lane 4) in comparison to transfected untreated cells (lane 3). Immunoprecipitation (IP) with anti-EE mAb from diamide-treated untransfected cells (lane 2) and Protein G beads coupled with anti-EE (lane 1) were used as a negative control. The expression of EE-p70S6K1 was examined by WB analysis with anti-S6K1 (C-terminal) antibody (lanes 3–6). Lane 7—protein markers. (**B**) CoAlation of EE-p70S6K1 in HEK293/Pank1β cells treated with diamide is shown by WB analysis of the same samples as in (**A**) using a near-infrared fluorescence detection system. The level of immunoprecipitated EE-p70S6K1 is shown in green and CoAlated proteins in red. Yellow colour demonstrates the overlap of fluorescent signals between the anti-CoA and anti-S6K1 immunoreactivities which corresponds to CoAlated EE-p70S6K1. The arrows indicate the position of EE-p70S6K1. The experiment was repeated three times with similar results obtained.

The identification of CoAlated p70S6K1 by MS-based methodology in diamide-treated HEK293/Pank1β cells prompted us to advance this finding. As shown in Figure 2, the treatment of transfected cells with diamide induces the readily detected covalent modification of cellular proteins by CoA in total cell lysates (lane 6), when compared to untreated cells (lane 5). Immunoprecipitated EE-S6K1 was found to be CoAlated in diamide-treated (lane 4), comparing to untreated transfected cells (lane 3). WB analysis with anti-S6K1 (C-terminal) polyclonal antibody (lanes 3–6) was used to examine the expression of EE-S6K1 (lanes 5 and 6) and the efficiency of its immunoprecipitation (lanes 3 and 4). Immunoprecipitation with anti-EE mAb from diamide-treated untransfected cells (lane 2) and Protein G beads coupled with anti-EE (lane 1) was used as a negative control. To validate the CoAlation of EE-p70S6K1 in HEK293/Pank1β cells treated with diamide, the same set of samples was analysed using near-infrared fluorescence detection system. Figure 2B shows the level of immunoprecipitated EE-p70S6K1 (in green) and CoAlated proteins (in red). The overlap of fluorescent signals (in yellow) between the anti-CoA and anti-S6K1 immunoreactivities corresponds to CoAlated EE-p70S6K1.

### 2.3. In Vitro CoAlation of Recombinant p70S6K1

Further, we examined whether recombinant p70S6K1 is CoAlated in vitro. The constitutively active form of p70S6K1 (His-actS6K1) produced by the Dual Bac-to-Bac protein expression system was used in this study.

**Figure 3:**
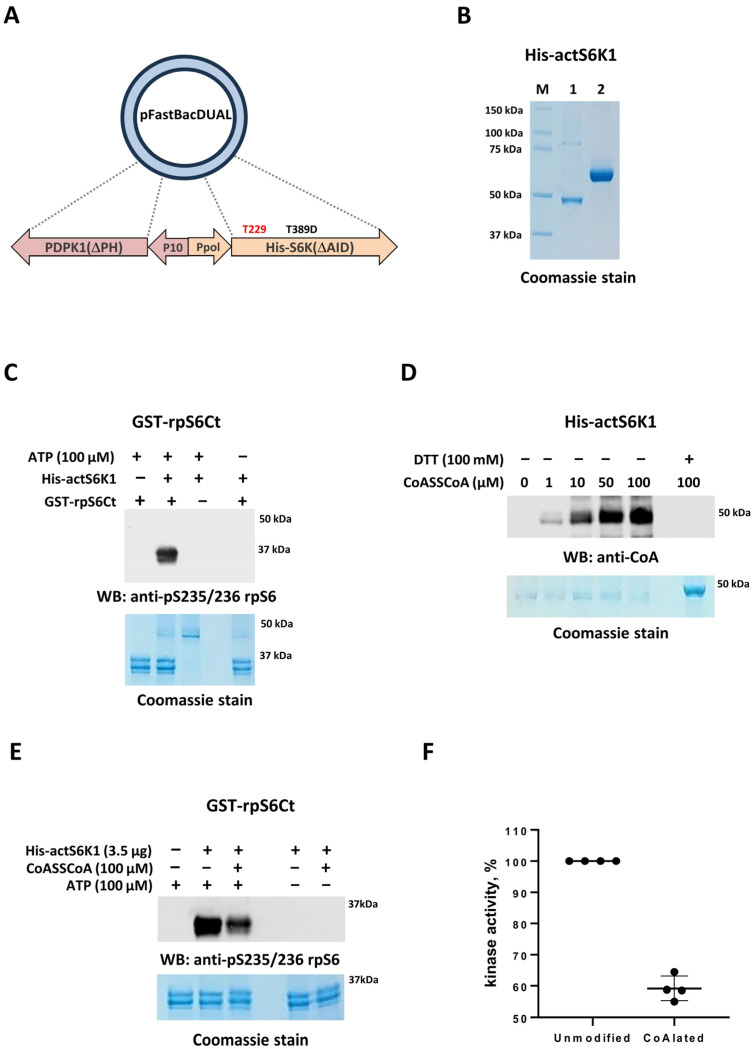
In vitro CoAlation of recombinant p70S6K1 and its in vitro kinase activity. (**A**) Schematic diagram of the of the bacmid construct expressing His-actS6K1. (**B**) SDS-PAGE analysis of the purified His-actS6K1 protein (Coomassie staining): PM—protein markers; 1—purified His-actS6K1 (1µg); 2—BSA (2µg). (**C**) Recombinant His-actS6K1 is active as shown by efficient phosphorylation of its physiological substrate, ribosomal S6 protein (anti-pS235/235 rpS6 Western blot). (**D**) In vitro CoAlation of His-actS6K1. (**E**) The activity of CoAlated His-actS6K1 is inhibited in vitro. (**F**) Results of densitometry analysis of in vitro kinase activity of CoAlated and control His-actS6K1 (four independent experiments). The quantitative analysis was performed using ImageJ software, and an unpaired Student’s t-test was used for statistical analysis, *p* < 0.01. The statistical analysis was carried out in GraphPad Prism (v10.2.0, GraphPad Software, Inc., Boston, MA, USA), and SD was used.

Briefly, using the pFastBac™ Dual vector, we created a recombinant plasmid co-expressing His-actS6K1 (lacking autoinhibitory C terminal domain and carrying Thr389E phosphomimicking mutation) along with ∆PHPDK1 (upstream regulator of Thr229 site of S6K1). The co-expression of ∆PHPDK1 from the dual expression system allows the phosphorylation of S6K1 at Thr229, leading to the constitutive activation of the recombinant kinase. The schematic diagram of the bacmid construct expressing His-actS6K1 is shown in Figure 3A. Sf9 insect cells were infected with the plasmid carrying virus and harvested after 48 h. The recombinant protein was then affinity purified from cell pellets using Ni-NTA agarose (Figure 3B), which resulted in a great yield of highly pure recombinant His-actS6K1 protein, with a molecular weight of ~45 kDa. The activity of purified His-actS6K1 was tested in vitro using the C-terminal region of ribosomal S6 protein fused with GST (GST-rpS6Ct) as a substrate. The phosphorylation of GST-rpS6Ct was monitored by Western blot analysis and was detected using the phospho-ribosomal protein S6 (pS235/pS236) antibody. As shown in Figure 3C, recombinant His-actS6K1 is constitutively active as the phosphorylation of GST-rpS6Ct at S235/S236 is readily detected in the presence of ATP. The specificity of this antibody towards phosphorylated GST-rpS6Ct was confirmed with an in vitro kinase assay performed without ATP.

We recently developed an efficient in vitro CoAlation assay which accurately detects the covalent CoA modification of purified recombinant and endogenous proteins [25]. Using this assay, we showed that CoAlation of His-actS6K1 occurs in a dose-dependent manner with an increasing concentration of CoA (Figure 3D). The presence of 100 mM DTT in the reaction mix completely abolishes His-actS6K1 CoAlation, confirming the disulfide bond formation between oxidized cysteine residue(s) and the CoA thiol group.

### 2.4. In Vitro Kinase Activity of CoAlated Recombinant p70S6K1

To test the effect of CoAlation on S6K1 activity, recombinant His-actS6K1 was CoAlated in vitro. The efficient CoAlation of His-actS6K1 was confirmed by immunoblotting with an anti-CoA antibody (Figure 3D). The kinase activity of CoAlated or control, unmodified His-actS6K1 samples towards GST-rpS6Ct was then measured by immunoblotting with the phospho-ribosomal protein S6 (pS235/pS236) antibody (Figure 3E). Figure 3F demonstrates that the in vitro CoAlation of His-actS6K1 results in a significant inhibition of the kinase activity (~40%), when compared to the unmodified control. These data indicate that the covalent modification of catalytically active His-actS6K1 by CoA results in the inhibition of its kinase activity.

### 2.5. Molecular Docking of CoA in the Crystal Structure of p70S6K1

**Figure 4:**
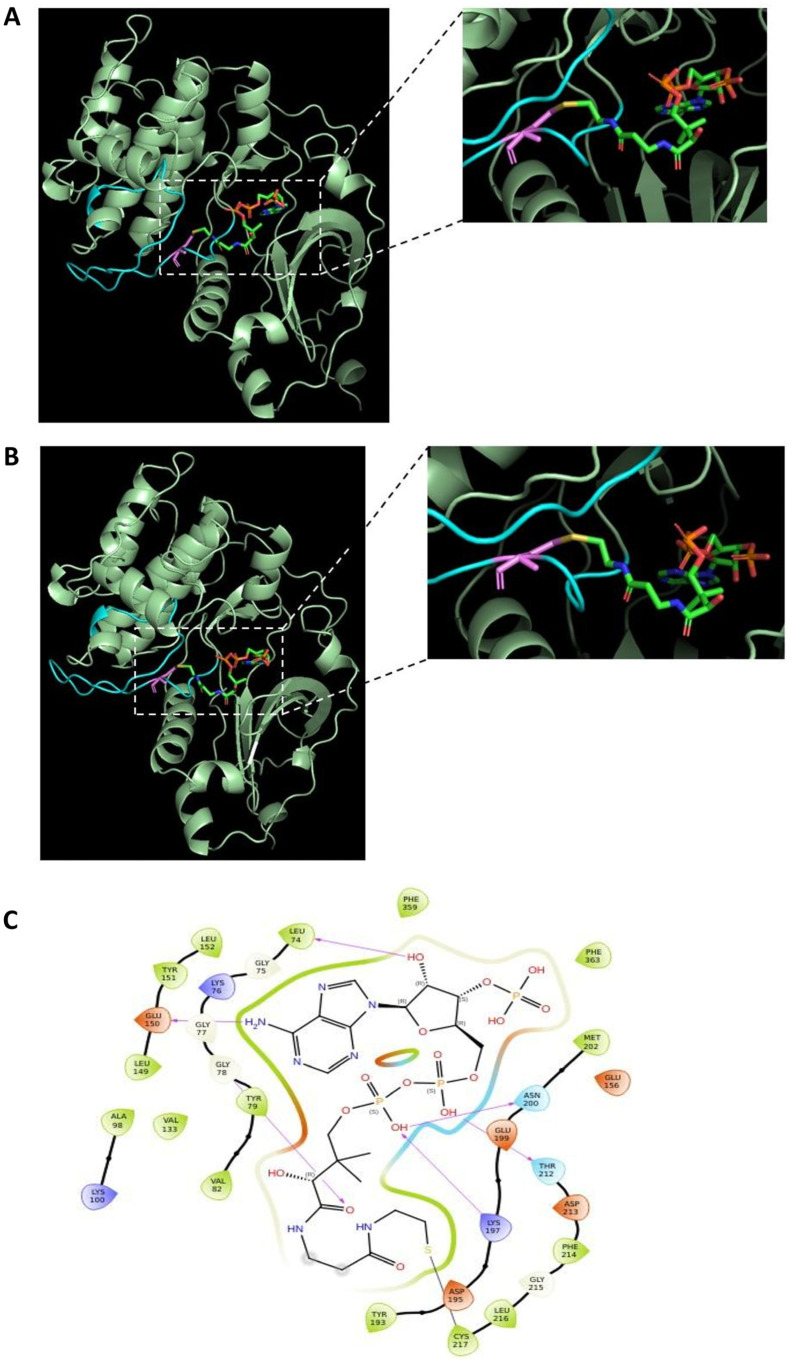
CoA binds covalently to Cys217 of p70S6K1. (**A**) Molecular docking of CoA in the 4L46 crystal structure of p70S6K1 and (**B**) phosphorylation at Thr229 and Thr389 in the active model form of p70S6K1. Missing residues in the p70S6K1 structure were modelled using the Swiss-Model web server. The close-up view shows the covalent binding of CoA (highlighted in red, green, blue, and yellow colours) to Cys217 (violet) in the activation loop (cyan) of p70S6K1 (pale green). (**C**) Diagram showing the pocket residues that interact with CoA. The purple arrows indicate hydrogen bonding interactions whereas the black line indicates covalent bond formation. The distance between the CoA S atom and S atom of Cys217 in both p70S6K1 forms were found to be 2.151 Å.

Figure 4A illustrates the crystal structure of the human p70S6K1 kinase domain plus the hydrophobic motif (4L46), which adopts a typical bilobal architecture with the ATP binding region formed between the N-terminal and C-terminal lobes. The protein was crystallised with the inhibitor 2-{[4-(5-ethylpyrimidin-4-yl) piperazin-1-yl] methyl}-5-(trifluoromethyl)-1H-benzimida-zole (5FI), which competes for the kinase ATP binding region [31]. The p70S6K1 crystal structure contains missing residues which were modelled using the Swiss-Model web server.

The covalent docking of the CoA thiol groups with Cys217 of the unphosphorylated and phosphorylated forms of p70S6K1 (Figure 4A,B, respectively) were carried out using the CovDock module in the Pose Prediction (Thorough) docking mode in the Schrödinger 2023 suite. The best pose obtained had a docking score of -9.796. The covalently docked pose matched with the superimposed pose, where the adenine ring is pointed towards the hinge region. The interactions of CoA with the protein in the best pose is illustrated in Figure 4C. The purple arrows indicate potential hydrogen bonding interactions, whereas the black line indicates the covalent bond formed. The distance between the sulphur atom of CoA and the sulphur atom of Cys217 is 2.151 Å. The stability of the best pose was checked by MD simulations for both unphosphorylated and phosphorylated (Thr229 and Thr389 sites) forms of p70S6K1.

The stability of CoA in the ATP-binding pocket of p70S6K1 was maintained by the 6-amino group of the CoA adenosine ring binding to the hinge region of S6K1 through a direct hydrogen bond with the main-chain carbonyl group of the Glu150 residue (hydrogen bond distance of 2.87 Å). The planar adenosine ring of CoA is involved in numerous hydrophobic interactions with residues Leu74, Val82, Ala98, Val133, Leu149, Leu152, Met202, Thr212, and Phe359.

### 2.6. Molecular Dynamics Simulations of the Complex

**Figure 5:**
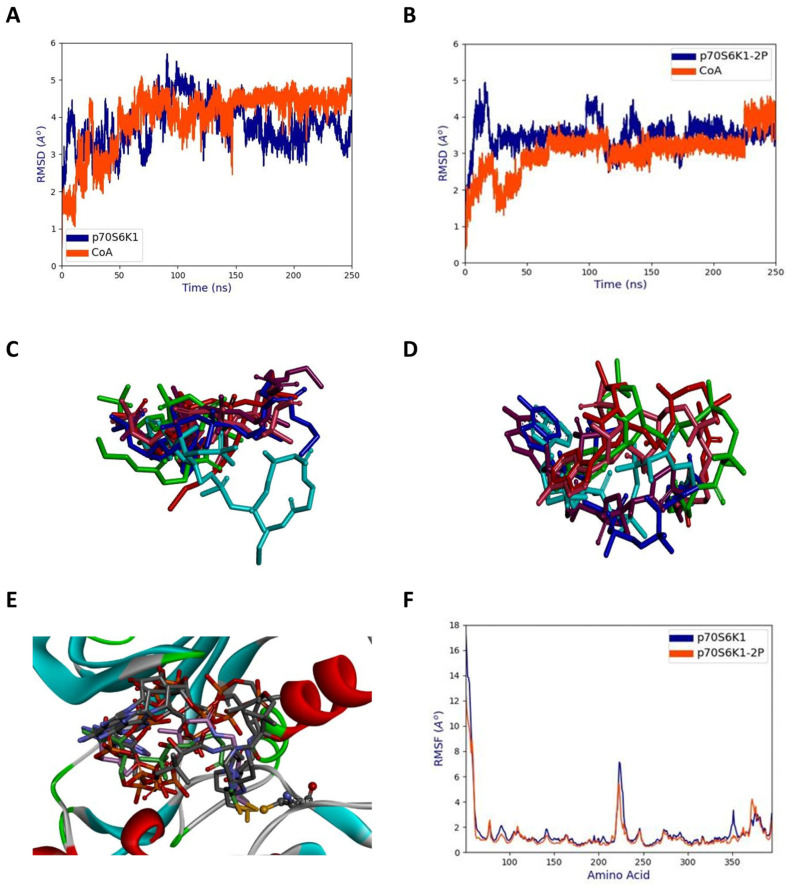
Molecular dynamics simulations of the p70S6K1-CoA complex. (**A**) The RMSD with respect to p70S6K1 (blue) and CoA (red) from MD simulations. (**B**) RMSD with respect to p70S6K1-2P (phosphorylated Thr229 and Thr389; blue) and CoA (red) from MD simulations. (**C**) Superposed poses of the docked conformation and snapshots of CoA at every 50 ns in the p70S6K1-CoA complex during MD simulations. Original docked pose (cyan), 50 ns (maroon), 100 ns (blue), 150 ns (pink), 200 ns (red), and 250 ns (green). (**D**) Superimposed poses of the docked conformation and snapshots of CoA at every 50 ns in the p70S6K1-2P (phosphorylated Thr229 and Thr389)–CoA complex during MD simulations. Original docked pose (cyan), 50 ns (maroon), 100 ns (blue), 150 ns (pink), 200 ns (red), and 250 ns (green). (**E**) Close-up view of CoA at every 50 ns in p70S6K1-2P (phosphorylated Thr229 and Thr389)–CoA complex during MD simulations. The Cys217 side chain is indicated in ball and stick notation and remains close to the CoA S atom. (**F**) RMSF plots for the p70S6K1-CoA (blue) and p70S6K1-2P (phosphorylated Thr229 and Thr389) (red) complex with CoA.

The phosphorylated and unphosphorylated forms of p70S6K1 complexed with the best pose of CoA were subjected to 250 ns of MD simulations using the Amber22 suite program. From the stabilized structures, it can be seen that the unphosphorylated (Figure 5A) form displayed higher structural deviations when compared to the phosphorylated form (Figure 5B). Similarly, CoA displayed a lower RMSD (Figure 5A) in the p70S6K1-2P complex (Figure 5B). This suggests that the interaction between CoA and the phosphorylated form of the kinase is more stable than with the unphosphorylated form. However, for both systems, the adenine ring is pointed towards the hinge region throughout the simulations. In the unphosphorylated form, it was found from the initial stages of MD simulations that the pantetheine tail of CoA moved away from the initial docking pose and had a variable location, indicating its flexibility but remaining within the binding cavity of p70S6K1 (Figure 5C). In contrast, for the phosphorylated form of the protein, the pantetheine tail of CoA moved closer to the p-loop and the αC-helix but was less flexible (Figure 5D) compared to the complex with the unphosphorylated kinase. Interestingly, in the complex with p70S6K1-2P the S atom of CoA remained close to the S atom of Cys217 throughout the MD simulations (Figure 5E). Hence, this suggests that the activated form of p70S6K1 is able to form stable S-S interactions with CoA, while such interactions would not occur with unphosphorylated form of the enzyme. The 220–226 region in the kinase activation loop showed higher fluctuations in both p70S6K1 and p70S6K1-2P forms of the protein (Figure 5F).

## 3. Discussion and Conclusions

The growth factor-mediated activation of p70S6K1 involves a series of phosphorylation events at the C-terminal autoinhibitory domain, which promotes its interaction and phosphorylation by mTOR and PDK1. In its activated state, p70S6K1 is implicated in the phosphorylation and regulation of a diverse range of substrates involved in transcription, protein synthesis, apoptosis, cell growth, and energy homeostasis. The dysregulation of p70S6K1 has therefore been linked to multiple pathologies, including aging, cancer, and metabolic disorders. These findings highlight p70S6K1 as an attractive drug target for therapeutic intervention.

The activity and function of p70S6K1 is regulated by other post-translational modifications, such as acetylation, ubiquitination, and O-GlcNAcylation. The C-terminal acetylation of p70S6K1 was shown to block Thr389 phosphorylation by mTOR, preventing its activation [32]. Moreover, SIRT1 and SIRT2 deacetylases prevented this inhibitory effect, indicating a cross-talk between mTOR/p70S6K1 and sirtuin pathways. The ubiquitination of p70S6K1 was observed in cells and was shown to control its steady-state level via the proteasome-mediated pathway [33]. This regulatory event was independent from the mitogen-induced phosphorylation/activation of the kinase [34]. The induction of p70S6K1 O-GlcNAcylation in cells by overnutrition results in the suppression of the mTOR/S6K1 pathway and the inhibition of macrophage proinflammatory signalling [35].

In this study, we report for the first time that oxidative stress induces covalent modification of p70S6K1 at Cys217 by CoA, which is well known for its function in cellular metabolism and the regulation of gene expression. This covalent modification caught our attention, because Cys217 is located in the kinase activation loop. Here we provide evidence that the CoAlation of p70S6K1 occurs following exposure to oxidative stress in vivo and in vitro and has an inhibitory effect on kinase activity. Moreover, the CoAlation of p70S6K1 under oxidative stress may serve other regulatory purposes, such as protection of Cys217 from irreversible over-oxidation, which could result in the loss of function and subsequent degradation of the protein. In addition, the covalent binding of the bulky CoA moiety to Cys217 may modulate the phosphorylation status of Thr229 by sterically hindering kinase access to this phosphorylation site, thereby regulating the interaction with PDK1 or Ser/Thr phosphatase.

Taking into account the existence of an additional Cys231 located next to Thr229 within the kinase activation loop, it cannot be excluded that the CoAlation of Cys217 might function as a safeguard against the formation of an intramolecular disulfide bond during oxidative stress, which, as shown for the AKT, MELK, and BRSK1/2 kinases, can lead to a complete inactivation of the kinase [36,37,38,39]. This is in contrast to the 40% decrease in kinase activity following the CoAlation of p70S6K1, suggesting that this post-translational modification may allow the fine-tuning of the response of kinases to oxidative stress. Finally, p70S6K1 CoAlation may facilitate redox signalling by creating a novel binding platform for antioxidant proteins possessing the Rossmann binding fold. Addressing these and other relevant research questions should remain the focus of further investigations.

A recent study from our laboratory revealed a unique mode of Aurora A kinase inhibition by CoA, which we termed the “dual anchor” mechanism [29]. It locks Aurora A kinase in an inactive state by the selective anchoring of the ADP moiety of CoA into the ATP binding pocket which allows the thiol group of the flexible pantetheine tail to covalently modify Cys290 in the activation loop. In subsequent studies, we demonstrated that metastasis suppressor protein NME1 is a major CoA-binding protein, and its nucleoside diphosphate kinase activity is inhibited via covalent or non-covalent CoA interactions [40]. Following these original findings, the crystal structure of hNME1 in complex with CoA was solved and revealed the mode by which CoA non-covalently interacts with the nucleotide-binding pocket of NME1 [41].

Both crystallographic studies showed that CoA binding to Aurora A kinase and NME1 differs significantly from that of ATP. The γ-phosphate of ATP is projected toward the catalytic site of both kinases, while the β-phosphate of CoA and the pantetheine tail are solvent exposed and oriented away from the catalytic pocket.

In the present study, using molecular docking and molecular dynamics simulations, we investigated the molecular interaction of CoA in the complexes with the unphosphorylated and phosphorylated (Thr229 and Thr389) forms of the p70S6K1 kinase. The formation of a H-bond between the NH_2_ group of the CoA adenosine ring and the main-chain glutamic acid residue of S6K, in addition to many hydrophobic interactions, stabilizes the adenine ring inside the S6K1 ATP-binding pocket for both phosphorylated and unphosphorylated complexes. Such an interaction resembles the dual-anchor mechanism of Aurora A kinase binding to CoA, as described previously.

During MD simulations, we observed the model of activated form of p70S6K1 to build up stable S-S interactions in complex with CoA, while at the same time such interactions were not witnessed in the complex with unphosphorylated form of the enzyme. Based on such observations, it was noticed that the CoA pantetheine tail remained close to Cys217 in the activation loop of the phosphorylated kinase throughout the duration of the MD simulations, while its ADP moiety was steadily oriented towards the kinase’s hinge region. According to these findings, we hypothesize that the kinase hinge mobility of p70S6K1-2P enables CoA to stabilize the covalent disulfide bond with Cys217 in the activation loop, compared to the unphosphorylated form of kinase.

In conclusion, we believe that the regulation of p70S6K1 activity by CoAlation could serve as alternative phosphatase-independent mechanism, playing an integral part in the cellular response to oxidative stress.

## 4. Materials and Methods

### 4.1. Reagents and Chemicals

Unless otherwise stated, all common reagents and chemicals were obtained from Sigma-Aldrich (Merck Life Science UK Limited, Dorset, UK), including coenzyme A sodium salt (CoASH), coenzyme A oxidized lithium salt (CoASSCoA), hydrogen peroxide (H_2_O_2_), diamide, N-ethylmaleimide (NEM), ATP, DTT, β-glycerophosphate, sodium dodecyl sulphate (SDS), and 1M Tris hydrochloride pH7.5. The cOmplete™ Protease Inhibitor Cocktail EDTA-free (PIC) was from Roche (#11836170001, Roche Diagnostics GmbH, Mannheim, Germany) and the protein marker was from BioRad (Precision Plus Protein Dual Color Standards, #1610374, Bio-Rad Laboratories, Inc. Watford, UK). InstantBlue^®^ Coomassie Protein Stain was obtained from Abcam (Abcam Limited, Cambridge, UK). The generation and characterization of the mouse anti-CoA monoclonal antibody 1F10 [27] (WB dilution 1:6000) as well as rabbit polyclonal anti-S6K1 C-terminal antibodies (WB dilution 1:4000) have been previously described [42]. The mouse anti-EE antibody used (WB dilution 1:6000) was generated, characterized, and generously supplied by Professor Peter Parker (King’s College London and The Francis Crick Institute); anti-pS235/236 rpS6—Phospho-S6 Ribosomal Protein Antibody was purchased from Cell Signaling Technology (WB dilution 1:5000, #2211, Cell Signaling Technology, Inc., Boston, MA, USA). Among the other antibodies used were Alexa Fluor 680 goat anti-mouse IgG H&L (WB dilution 1:10,000, #A21057, Invitrogen, Thermo Fisher Scientific Inc., Waltham, MA, USA) and IRdye 800 CW goat anti-rabbit IgG H&L (WB dilution 1:10,000, #A32735, Invitrogen, Thermo Fisher Scientific Inc., Waltham, MA, USA). Primary antibodies were diluted in Odyssey blocking buffer containing 0.01% Tween 20. Secondary antibodies were diluted in Odyssey blocking buffer (WB dilution 1:10,000) containing 0.02% SDS.

### 4.2. Mammalian Cell Culture

All cell-based experiments were performed using human embryonic kidney 293 (HEK293) cells stably overexpressing the rate-limiting enzyme in CoA biosynthesis, pantothenate kinase 1β (Pank1β). The generation of the HEK293/Pank1β cell line was previously reported [25]. HEK293/Pank1β cells were cultured in Dulbecco’s Modified Eagle Medium (DMEM) (Lonza, SLS, Nottingham, UK) supplemented with 10% foetal bovine serum (FBS) (HyClone, SLS, Nottingham, UK), 50 units/mL penicillin and 50 µg/mL streptomycin (Lonza, SLS, Nottingham, UK) at 37 °C in the presence of 5% CO_2_. The induction of oxidative stress was conducted by treating the cells with 0.5 mM H_2_O_2_ or 0.5 mM diamide for 30 min. Prior to use, cells were tested and shown to be free of mycoplasma infection.

### 4.3. Mass Spectrometry and Data Processing

Mass spectrometry data acquisition and analysis were performed as described by Y. Tsuchiya et al. [25]. Briefly, the S6K1-derived peptides were immunoprecipitated with an anti-CoA antibody from trypsin/LysC digested protein lysates of HEK293/Pank1β cells treated with 500 µM diamide. After the treatment with Nudix 7, immunoprecipitated peptides were further enriched using an IMAC column. The reaction mix was analysed by nano-scale capillary liquid chromatography–tandem mass spectroscopy (LC-MS/MS) using an Ultimate U3000 HPLC (ThermoScientific Dionex, San Jose, CA, USA) and C18 Acclaim PepMap100 5 µm and C18 Acclaim PepMap100 3 µm matrixes. Peptides were eluted with a gradient of acetonitrile and analysed by an ion trap mass spectrometer (Orbitrap Velos, ThermoScientific, San Jose, CA, USA). Data-dependent analysis was carried out, using a resolution of 60,000 for the full MS spectrum, followed by 20 MS/MS spectra in the linear ion trap. MS/MS scans were collected using an automatic gain control value of 1  ×  10^4^ and a threshold energy of 35 for collision-induced dissociation.

LC–MS/MS raw data files were processed using MaxQuant [43] version 1.5.2.8, which incorporates the Andromeda search engine. For all datasets, the default parameters in MaxQuant were used, except for MS/MS tolerance at 0.6 Da and the second peptide ID was unselected. The CoAlation of cysteine with delta mass values 338, 356, and 765 were allowed as variable modifications. MaxQuant-processed data were searched against a protein sequence database (Uniprot KB-human, June 2013) database.

Using MQ viewer, CoA_356 peptides were first visually checked and those MS/MS scans that ‘failed’ visually were checked manually. The corresponding MS/MS scan was manually sequenced to match the identified peptide.

### 4.4. Transient Transfection and Immunoprecipitation

HEK293/Pank1β cells were seeded onto 6 cm Petri dishes and transfected at 50% confluence with pcDNA3.1/EE-p70S6K1 vector encoding EE-tagged full-length human p70S6K1 using TurboFect transfection reagent (ThermoScientific Dionex, San Jose, CA, USA), according to the manufacturer’s protocols. After 24 h, the culture medium was replaced with pyruvate-free DMEM supplemented with 5 mM glucose and 10% FBS. After an 18 h incubation, cells were treated with 500 μM diamide for 30 min at 37 °C, harvested by pressure washing, and centrifuged at 1800× *g* for 5 min at RT. Harvested cells were lysed at 4 °C for 20 min in lysis buffer, containing 50 mM Tris-HCl pH 7.5, 150 mM NaCl, 5 mM EDTA, 50 mM NaF, 5 mM Na_4_P_2_O_7_, and 1% Triton X-100, supplemented with 100 mM NEM, protease and phosphatase inhibitors cocktails. Total cell lysates were centrifuged at 20,817× *g* for 20 min at 4 °C, and the supernatant was collected for further analysis. Protein concentration was measured using the Thermo Scientific Pierce Coomassie Protein Assay Kit (#23200, Pierce). The immunoprecipitation of transiently expressed EE-tagged p70S6K1 from cell lysates was carried out using Protein G Sepharose (Generon, Slough, UK) and an anti-EE tag monoclonal antibody. Proteins were eluted from beads with 2× SDS loading buffer without DTT (non-reducing condition), boiled for 5 min, and analysed by WB using anti-CoA and anti-S6K1 C-terminal antibodies.

### 4.5. Western Blot Analysis

Prepared samples were separated by SDS-PAGE in a 4–20% Precast Gel (Merck Life Science UK Limited, Dorset, UK) and transferred to a low-fluorescence PVDF membrane (Millipore, Merck Life Science UK Limited, Dorset, UK), which was then blocked with Intercept^®^ (TBS) Protein-Free blocking buffer (LI-COR Biosciences, LI-COR Biosciences UK Ltd., Cambridge, UK) for 30 min. The membrane was incubated with primary antibodies for an hour at room temperature (RT) or overnight at 4 °C. The membrane was then washed extensively with Tris-buffer saline supplemented with 0.05% Tween 20 (TBSTw) before incubation with the secondary antibodies for 30 min at RT. Immunoreactive bands were visualised using Odyssey Scanner CLx and Image Studio Lite software version 5.0 (LI-COR Biosciences, LI-COR Biosciences UK Ltd., Cambridge, UK).

### 4.6. Insect Cell Culture

Sf9 (*Spodoptera frugiperda*) insect cells were cultured as suspension cultures in Erlenmeyer flasks (1000 mL) with constant shaking at +27 °C in Insect-XPRESS™ Medium (Lonza, SLS, Nottingham, UK), supplemented with 10% foetal bovine serum (FBS) and 1% antibiotics (penicillin and streptomycin).

### 4.7. Sf9 Cells Infection and Expression of Recombinant His-actp70S6K1

Recombinant bacmid DNA was generated using the Dual Bac-to-Bac system as described elsewhere. The plasmid containing His-actp70S6K1 and ∆PHPDK1 was used for the infection of Sf9 insect cells. Briefly, the bacmid DNA was transfected into Sf9 insect cells using CellFECTIN (ThermoScientific Dionex, San Jose, CA, USA). Three days after transfection, the V_0_ virus was harvested and used to produce a V_1_ virus stock, which in turn was used to infect 500 mL Sf9 cells for protein expression. After infection, cells were monitored and harvested by centrifugation. Cell pellets were washed with PBS, flash-frozen in liquid nitrogen, and stored at −80 °C until purification.

### 4.8. Purification of the Constitutively Active Recombinant His-actS6K1 Protein

Recombinant His-actp70S6K1 protein was purified using Ni-NTA affinity chromatography as described. Briefly, the cell pellet of baculovirus infected Sf9 cells was resuspended in ice-cold lysis buffer of 20 mM Tris–HCl (pH 8.0), 200 mM NaCl, 2 mM MgCl_2_, 2 mM NaF, 10% glycerol, 10 mM imidazole, 1% Nonidet P40, 1 mM PMSF, 50 mM β-glycerophosphate, 1xPIC EDTA free, 25 U/mL benzonaze, and lysed on ice for 30 min with periodic vortexing. After centrifugation for 30 min at 14,000 rpm, the cell supernatant containing His-actp70S6K1 was collected and loaded onto the pre-equilibrated Ni-NTA affinity resin (with 20 mM Tris–HCl pH 8.0, 200 mM NaCl) (#NB-45-00042-100, Neo Biotech, Generon, Slough, UK). The incubation was carried out for 2 h under constant slow rotation, and then the column was washed 5 times with a washing buffer containing 20 mM Tris–HCl (pH 8.0), 200 mM NaCl, and 20 mM imidazole. The elution of bound proteins was performed with buffer containing 20 mM Tris–HCl (pH 8.0), 200 mM NaCl, and 350 mM imidazole. The two-step dialysis of purified recombinant protein was conducted against 20 mM Tris–HCl (pH 7.5), 200 mM NaCl and 50% glycerol buffer using Spectra/Por^®^ 3 dialysis membrane with a MW cutoff 3.5 kDa (ThermoScientific Dionex, San Jose, CA, USA).

### 4.9. In Vitro CoAlation Assay

For the in vitro CoAlation assay developed previously [25], 1.5 μg of purified recombinant His-actS6K1 protein was incubated with the oxidized form of 100 μM CoA (CoASSCoA) in 20 mM Tris-HCl, 100mM NaCl, and pH 7.5 for 30 min at RT. The reaction was stopped by adding SDS gel-loading buffer without DTT but containing 100 mM NEM. To confirm the specificity of the reaction, 100 mM DTT was added to another reaction mixture.

### 4.10. In Vitro Kinase Assay

The in vitro kinase assay was performed at 37 °C for 30 min in an Eppendorf thermomixer, using 1 µg of recombinant His-actS6K1, 1 µg of recombinant GST-rpS6(Ct) as a kinase substrate, and 100 μM ATP in 20 µL of kinase reaction buffer (25 mM Tris-HCl pH 8.0, 10 mM MgCl2, 5 mM β-glycerophosphate). The reaction was stopped by the addition of 5x Laemmli buffer. Samples lacking recombinant kinase or ATP were used as negative controls. In vitro CoAlation was carried out in the presence of CoA dimer (CoASSCoA) as described above. CoAlated His-actp70S6K1 was used in the in vitro S6 kinase assay, and the outcome of reaction was visualized by WB analysis using an anti-pS235/236 rpS6—Phospho-S6 Ribosomal Protein Antibody. The quantitative analysis was performed using ImageJ software, and an unpaired Student’s t-test was used for statistical analysis, *p* < 0.01. The results of the 4 independent experiments of the kinase activity are summarized as mean ± standard deviation. Statistical analysis was performed in GraphPad Prism (v10.2.0, GraphPad Software, Inc., Boston, MA, USA).

### 4.11. Molecular Docking

Protein Preparation: The crystal structure of *Homo sapiens* p70S6K1 (PDB_ID: 4L46) was obtained from the protein data bank (RCSB) [44]. The crystal structure contained missing residues, which were built using Swiss-Model web server [45]. The modelled protein was prepared using the “protein preparation wizard” of Maestro, Version 13.8. This step adds hydrogen atoms to the model and optimizes their orientation in order to maximize the hydrogen bond network and minimize the structure to a local minimum using OPLS4 force field. The prepared protein coordinates were saved in .maegz format.

Ligand Preparation: The coordinates of CoA were obtained from the crystal structure of *Homo sapiens* Aurora-A kinase (PDB_ID: 6I2U). CoA was prepared using the LigPrep module in the Schrödinger 2023 suite. The LigPrep module adds hydrogen atoms to CoA and designates appropriate valencies for all the atoms. The prepared CoA coordinates were saved in .maegz format.

The best binding pose of the CoA to p70S6K1 is generated by molecular docking. The grid box was constructed to cover the active site residues. It was centred at −0.5, 1.4, and 3.5 Å at the x, y, and z coordinates. The covalent docking of CoA with a disulfide bridge between the S atom of CoA and the S atom of Cys217 was carried out using the CovDock module in Pose Prediction (Thorough) docking mode in the Schrödinger 2023 suite. CovDock works by Glide docking to a receptor with the reactive residue trimmed to alanine. After adding the receptor reactive residue, different poses are sampled in order to form a covalent bond with the ligand. One output pose was generated per ligand reaction site. The covalent docked pose of CoA was saved in pv.maegz format.

### 4.12. Molecular Dynamics Simulations

The MD simulations were performed using AMBER. The active form of p70S6K1 was obtained by phosphorylating the residues Thr229 and Thr389 through Discovery Studio Visualizer [31]. The phosphorylated and unphosphorylated forms of the protein complexed with the best docked pose of CoA obtained from covalent docking studies were subjected to 250 ns molecular dynamics (MD) simulations using Amber 22 suite [46]. The AMBERff14SB force field [47] was applied to the protein, and GAFF force field [48] was applied to CoA. The force field parameters for phosphorylated threonine [49] were loaded. The protein structure was checked using antechamber [50] and was protonated at biological pH using the H++ web server [51]. For CoA, the hydrogen atoms were added in UCSF Chimera [52], and the mol2 and frcmod files were generated using antechamber [50] and parmchk2, respectively. The complexes generated by the tleap module for both molecular systems were immersed in a TIP3P water cubic box [53] with a 10 Å distance between the protein and the edge of the box. Unbalanced charges in the complex were neutralised by adding counter ions (Cl^−^). The tleap command generated solvated topology and coordinate files for both molecular systems.

Before equilibration, a first round of minimization was carried out for 10,000 steps, restraining the protein atoms, using the steepest descent and conjugate gradient algorithm to remove short-range bad contacts. An unrestrained second round of minimization was carried out for 200 steps. The system was heated from 0 K to 300 K in the NVT ensemble over a period of 2 ns. After heating, the system was equilibrated in the NPT ensemble (P = 1atm) twice over a period of 1.5 ns. For temperature and pressure control, Langevin thermostat and Berendsen barostat [54] were used, respectively. The production run was carried out for 250 ns involving 125 million steps. Minimization, equilibration, and production runs were carried out using pmemd, the MD engine of AMBER. For both the equilibration and production run, integration time step was 2fs and the SHAKE algorithm [55] was used to constrain bonds between heavy atoms and hydrogen. The particle mesh Ewald method [56] was used to deal with long-range electrostatic interactions. The cpptraj module [57] generated RMSD-values with reference to Cα atoms of protein and CoA, RMSF-values, and the number of intermolecular hydrogen bonds between CoA and the protein. Interactive Python 7.18.1.was used for plotting the data.

## Data Availability

Data is contained within the article.
